# Case Report: Baricitinib as an Alternative in the Maintenance Therapy for Macrophage Activation Syndrome Secondary to Nodular Panniculitis

**DOI:** 10.3389/fimmu.2022.914265

**Published:** 2022-07-06

**Authors:** Guanqun Yi, Zhengping Huang, Zhixiang Huang, Yunqing Wang, Weiming Deng, Shaoling Zheng, Tianwang Li

**Affiliations:** ^1^ Department of Rheumatology and Immunology, Guangdong Second Provincial General Hospital, Guangzhou, China; ^2^ The Second School of Clinical Medicine, Southern Medical University, Guangzhou, China; ^3^ Department of Rheumatology and Immunology, Zhaoqing Central People’s Hospital, Zhaoqing, China

**Keywords:** baricitinib, macrophage activation syndrome, nodular panniculitis, maintenance therapy, case report

## Abstract

**Background:**

Macrophage activation syndrome (MAS) is a severe complication of autoimmune diseases with high mortality. We report the effectiveness of baricitinib as an option for the maintenance therapy in MAS secondary to nodular panniculitis.

**Case summary:**

A 24-year-old female came to our hospital with repeated fever and a skin nodule on right tibial tuberosity. Results were notable for raised serum ferritin (SF), triglycerides (TG), elevated liver function enzymes, interleukin-6 (IL-6), interferon-γ (IFN-γ), soluble interleukin-2 receptor (sIL-2R) and decreased activity of NK cells. The pathological biopsy of the subcutaneous nodules indicated nodular panniculitis. Hemophagocytic cells were found in bone marrow aspiration. She was diagnosed as MAS secondary to nodular panniculitis. With the treatment of methylprednisolone (MP) and immunoglobulin, her symptoms and laboratory data gradually improved. Nevertheless, her disease relapsed when the MP dose was tapered. Regarding the usage of JAK inhibitors in MAS, we used baricitinib (JAK1/2 inhibitor) to treat MAS and her symptom and abnormal laboratory findings returned to normal. During follow-up, though the MP dose was tapered, she was stable without a MAS recurrence.

**Conclusion:**

The case report suggested baricitinib is an option for MAS in the maintenance therapy phase and is potentially beneficial to prevent recurrence.

## Introduction

Nodular panniculitis is a rare, chronic and recurrent disease originating from fatty lobules. Subcutaneous nodules are the most commonly symptom, followed by fever and arthralgias (1, 2). MAS caused by excessive activation or proliferation of T lymphocytes and macrophages and characterized by production of a cytokine storm (3, 4), is a serious complication of autoimmune diseases with high mortality (5). In addition, multiple studies have reported that MAS is prone to relapse and refractory (6–8). Therefore, timely diagnosis and prompt medical treatment are essential to the prognosis of patients. Here we report a clinically rare case of MAS secondary to nodular panniculitis, in which baricitinib plays a potential role in the maintenance treatment and the prevention of recurrence. To our knowledge, this is the first report of the effectiveness of baricitinib as an option for maintenance treatment in MAS secondary to nodular panniculitis.

## Case Report

**Figure 1:**
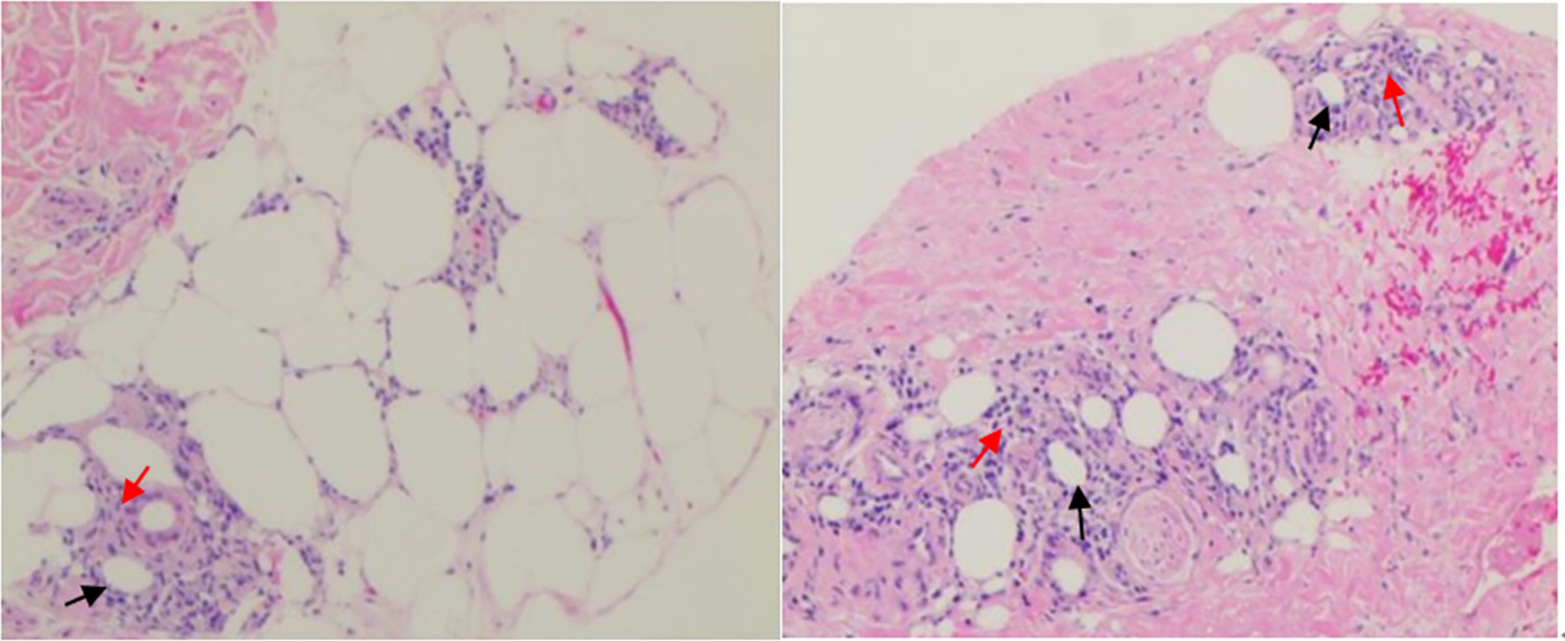
Biopsy specimens of cutaneous tubercle of the right tibia. Photograph showing local degeneration and necrosis of subcutaneous fat(↑), revealing lymphocytic infiltration(↑) with irregular nuclear morphology and nuclear rupture in the fat lobules, being in line with changes in nodular panniculitis. Specific stain: Periodic Acid Schiff Diastase (PAS-D) (-), Alcian blue (AB) (-). Direct immunofluorescence assay: IgG, C3, IgM, IgA (-).

**Figure 2:**
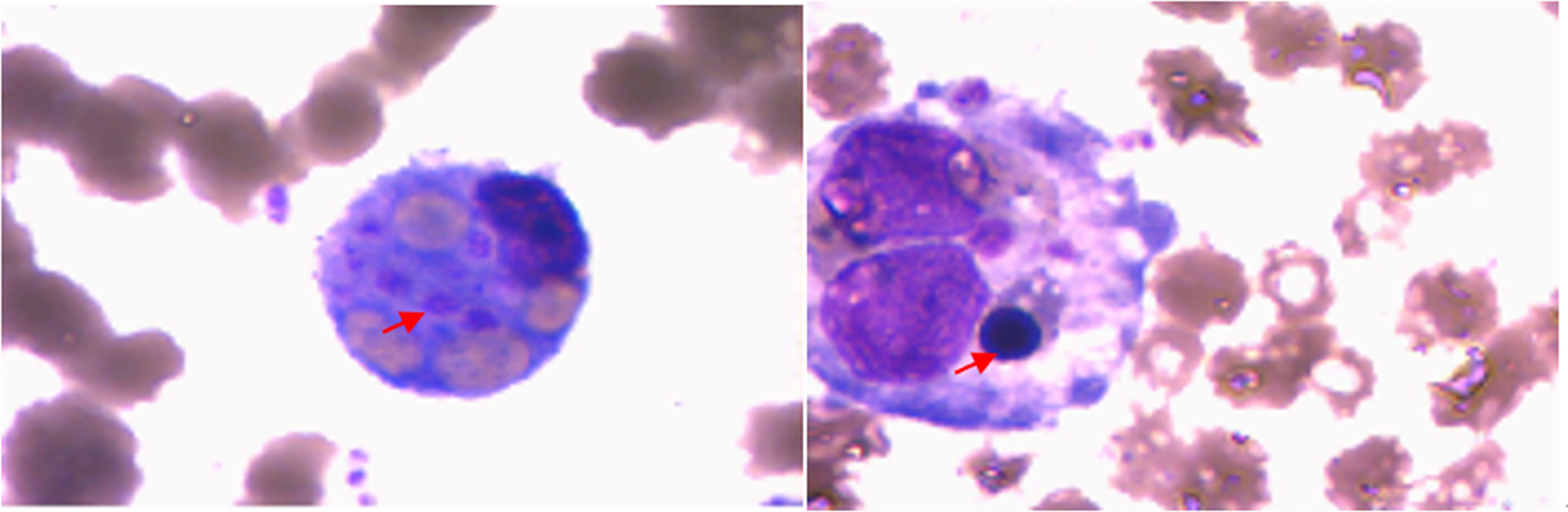
Bone marrow smears showing hematopoietic cells (↑) and increased reticulocytes.

A 24-year-old female patient presented to our hospital on June 10, 2020 with a 26-day history of repeated fever (Maximum temperature 39.5°C) and ankle arthritis. Skin nodule (one, 1 cm × 0.5 cm, tenderness) was at 5cm below the right tibial tuberosity. The family history was silent. Laboratory data during admission were notable for mild anemia, and raised SF, LDH, TG, elevated liver function enzymes, IL-6, IFN-γ and sIL-2R, as well as decreased activity of NK cells. Autoantibody profile (antinuclear antibody, antibodies to extractable nuclear antigen, anti-phospholipid antibody, rheumatoid factor, anti-cyclic citrullinated peptide antibody, and anti-neutrophil cytoplasmic autoantibody were all negative), thyroid function, infection indexes (procalcitonin, bacterial and fungal culture of bone marrow, and examinations of Cytomegalovirus and Epstein-Barr virus) and serum tumor markers were normal. Whole body PET-CT didn’t find any signs of tumor or abnormal lymph nodes. Ultrasound suggested normal liver and spleen. The pathological biopsy of the subcutaneous nodules indicated nodular panniculitis (**Figure 1**). Hemophagocytic cells were found in bone marrow aspiration (**Figure 2**). We didn’t see Reed-Sternberg cells or lymphoma cells in the bone marrow smear. Considering the clinical characteristics and results from the tests especially pathological biopsy findings from the subcutaneous nodules, we made the diagnosis of nodular panniculitis for the patient (9). Besides, although the patient didn’t fulfill the most commonly used criteria for hemophagocytic lymphohistiocytosis (HLH) (10), she met the reactive hemophagocytic syndrome diagnostic score (HScore) (11). Hence, she was considered as a rare case of MAS secondary to nodular panniculitis. She was treated with glucocorticoids (started with MP 40mg bid), immunoglobulin and cyclophosphamide (the cumulative dosage was 2.0g). Besides, methotrexate (MTX) and hydroxychloroquine (HCQ) were used for nodular panniculitis on June 28, 2020. Her symptoms and laboratory data improved. She was discharged with 20mg oral prednisone twice a day, 10mg MTX once a week and 0.2g HCQ twice a day.

**Figure 3:**
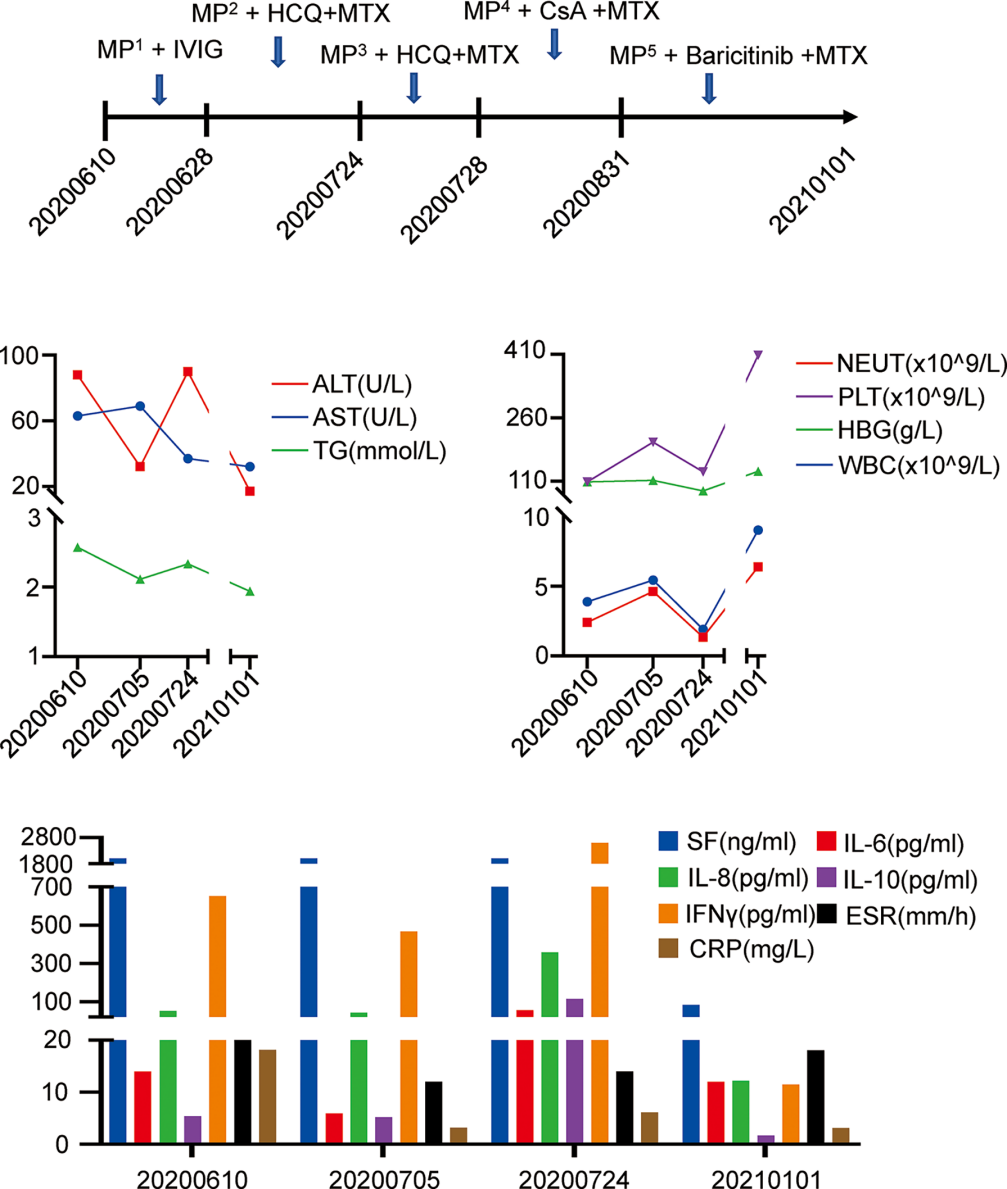
Clinical course and laboratory examination. IVIG, immu-noglobulin. (MP^1^ 80-40mg daily, MP^2^ 60-28mg daily, MP^3^ 500-40mg daily, MP^4^ 500-20mg daily, MP^5^ 20-1mg daily).

Nevertheless, fever recurred when the patient reduced the prednisolone dose to 20mg 8am and 15mg 4pm. She visited our department on July 24, 2020. Laboratory examination during the second admission showed cytopenia (including hemoglobin and neutrophils), hypofibrinogenemia, hypertriglyceridemia, hyperferritinemia and abnormal liver function tests. In addition, she had decreased NK cell activity, elevated sIL-2R and abnormal cytokine levels. Ultrasound suggested splenomegaly. She met the HScore as well as met six of eight clinical criteria for HLH. She was considered as a relapse of MAS secondary to nodular panniculitis. She was treated with glucocorticoids (MP 0.5g/d for 5 days), immunoglobulin and cyclosporine (CsA) on July 28, 2020. Her symptoms and laboratory abnormality improved gradually. She was discharged on August 12, 2020 with medications (prednisone 25mg bid and MTX 10 mg qw). Soon after the discharge, the patient required not to use CsA on August 31, 2020 due to her increased hair growth potentially caused by CsA. Therefore, CsA was switched to baricitinib 2mg daily with the permission of the patient on August 31, 2020. During one and a half years of follow-up, she is stable without a MAS recurrence, though the prednisolone dose is reduced to 5 mg every three days. The abnormal laboratory findings all returned to normal (**Figure 3**).

## Discussion

MAS secondary to nodular panniculitis is extremely rare. Only one case was reported from West China Hospital in 2018 (2). During the patient’s first visit, we made the timely MAS diagnosis based on HScore criteria, although the patient didn’t meet 2004 HLH criteria yet. The patient responded well to the treatment. However, her disease relapsed during the steroids tapering. Given the relapse, we increased steroids dose and added CsA to the patient. CsA is widely used to treat MAS. The patient’s symptoms overall improved with the treatment of high-dose steroids and CsA. But the patient refused CsA because of its side effect. There are two case reports in terms of the usage of JAK inhibitors in MAS. A trial demonstrated that ruxolitinib, a JAK1 and JAK2 inhibitor, is effective in improving cytopenias and inflammatory markers (SF, sIL-2R) and alleviating activation of T lymphocytes and monocytes, leading to discontinuation of corticosteroids and hospital discharge earlier (12). Another case reported that tofacitinib, a JAK1/2/3 inhibitor, induced remission in refractory adult-onset Still’s disease complicated by MAS (13). Baricitinib is another JAK1/2 inhibitor (14). Therefore, we used baricitinib (2mg, daily) to treat MAS instead of CsA. The patient’s symptoms improved and there is no MAS recurrence even with steroid reduction. Moreover, during the patient’s treatment with baricitinib and follow-up of one and a half years, there were no secondary infection or other related adverse reaction symptoms. The patient used CsA for approximately 1 month, which absorption half-life ranging from 0.5 to 2h (15). However, during the follow-up period of one and a half years after CsA suspension, the patient still successfully reduced steroids and no MAS recurrence to her. Therefore, we believe that baricitinib plays a potential role in the maintenance therapy for MAS secondary to nodular panniculitis.

In summary, we reported a rare case of recurrent MAS secondary to nodular panniculitis and for the first time found that baricitinib can be an option for MAS in the maintenance treatment phase. However, further study is warranted.

## Patient Perspectives

Throughout the clinical course, the patient and her parents were fully informed regarding the treatment and the potential side effects. They agreed the treatment.

## Data Availability Statement

The original contributions presented in the study are included in the article/Supplementary Material. Further inquiries can be directed to the corresponding authors.

## Ethics Statement

The studies involving human participants were reviewed and approved by the Ethics Committee of Guangdong Second Provincial General Hospital. The patients/participants provided their written informed consent to participate in this study. Written informed consent was obtained from the individual(s) for the publication of any potentially identifiable images or data included in this article.

## Author Contributions

GY actively wrote the manuscript and created the figures. ZPH provided constructive advice and critically revised the manuscript. ZXH edited the manuscript. YW, WD and SZ cared for the patient. TL guided the treatment of the patient. WD, SZ and TL took overall responsibility for the research performed in this study and for data integrity. All authors have read and approved the submitted version. All authors contributed to the article and approved the submitted version.

## Funding

This study was supported by Special fund of Guangdong Second Provincial General Hospital (Grant number: TJGC-2021004).

## Conflict of Interest

The authors declare that the research was conducted in the absence of any commercial or financial relationships that could be construed as a potential conflict of interest.

## Publisher’s Note

All claims expressed in this article are solely those of the authors and do not necessarily represent those of their affiliated organizations, or those of the publisher, the editors and the reviewers. Any product that may be evaluated in this article, or claim that may be made by its manufacturer, is not guaranteed or endorsed by the publisher.
